# Dried yeast cell walls high in beta-glucan and mannan-oligosaccharides positively affect microbial composition and activity in the canine gastrointestinal tract in vitro

**DOI:** 10.1093/jas/skaa173

**Published:** 2020-06-04

**Authors:** Pieter Van den Abbeele, Cindy Duysburgh, Maike Rakebrandt, Massimo Marzorati

**Affiliations:** 1 ProDigest bvba, Ghent, Belgium; 2 Leiber GmbH, Bramsche, Germany; 3 Center of Microbial Ecology and Technology (CMET), Ghent University, Ghent, Belgium

**Keywords:** dogs, in vitro, microbiota, propionate, *Saccharomyces cerevisiae*

## Abstract

The outer cell wall of yeast is characterized by high levels of β-glucans and mannan-oligosaccharides (MOS), which have been linked with beneficial effects on intestinal health and immune status in dogs. In this study, a standardized in vitro simulation of the canine gastrointestinal tract (Simulator of the Canine Intestinal Microbial Ecosystem; SCIME) was used to evaluate the effect of a *Saccharomyces cerevisiae*-based product, consisting of 27.5% β-glucans and 22.5% MOS, on the activity (as assessed by measurement of fermentative metabolites) and composition (as assessed by 16S-targeted Illumina sequencing) of canine intestinal microbiota. The *S. cerevisiae*-based product was tested at three different dosages, i.e., 0.5, 1.0, and 2.0 g/d. A dose-dependent fermentation pattern was observed along the entire length of the colon, as shown by the increased production of the health-related acetate, propionate, and butyrate for the three concentrations tested (0.5, 1.0, and 2.0 g/d). A consistent finding for all three tested concentrations was the increased propionate production (*P* < 0.05) in the simulated proximal and distal colon. These changes in terms of fermentative metabolites could be linked to specific microbial alterations at the family level, such as the specific stimulation of the propionate-producing families Porphyromonadaceae and Prevotellaceae upon in vitro exposure to the *S. cerevisiae*-based product. Other consistent changes in community composition upon repeated exposure included the decrease in the Enterobacteriaceae and the Fusobacteriaceae families, which both contain several potentially opportunistic pathogens. Altogether, the generated data support a possible health-promoting role of a product high in β-glucans and MOS when supplemented to the dogs’ diet.

## Introduction

Recently, advances in next-generation sequencing have enabled the characterization of the canine gut microbiome and revealed that it is highly influenced by the type of diet (Kim et al., 2017), indicating that dietary interventions might play a key role in canine health. Mannan-oligosaccharides (**MOS**), generally derived from the outer cell wall of *Saccharomyces cerevisiae*, have been a widely studied substrate in association with canine gut health (Swanson et al., 2002; Grieshop et al., 2004; Gouveia et al., 2006). The prebiotic properties of MOS are mainly attributed to their role in pathogenic resistance. Numerous pathogenic species contain mannose-specific type-1fimbriae which play a crucial role in their adherence to the gut wall, thereby stimulating the onset of infection. MOS act as a binding ligand to these type-1 fimbriae, resulting in the reduction of pathogenic colonization in the gastrointestinal tract (Oyofo et al., 1989; Spring et al., 2000). Indeed, Strickling et al. (2000) reported a reduction of the opportunistic pathogen *Clostridium perfringens* in the feces of dogs fed a diet supplemented with MOS. Similarly, Middelbos et al. (2007) showed a numerical reduction of *Escherichia coli* in the feces of adult dogs fed a yeast cell wall preparation high in MOS. Besides lowering the abundance of pathogenic species, supplementation of MOS has been shown to beneficially impact canine intestinal health by promoting the growth of *Lactobacillus* (Swanson et al., 2002) and *Bifidobacterium* (Strickling et al., 2000; Grieshop et al., 2004) species.

Next to high levels of MOS, the outer cell wall of *S. cerevisiae* is characterized by a high β-glucan content (Manners et al., 1973), which also contributes to canine gut health. Depending on the source, each β-glucan has a different type of linkage, branching structure, solubility, and molecular weight, which largely affect their functional properties (Brown and Gordon, 2005; El Khoury et al., 2012). The β-glucans derived from yeast typically contain a linear backbone of β-1,3-glycosidic linkages substituted with a limited amount of β-1,6-linked side chains (Manners et al., 1973; Brown and Gordon, 2005). Such β-glucans are known for their immunomodulatory activity and have already been shown to ameliorate immune status in dogs (Stuyven et al., 2010; Rychlik et al., 2013). Rychlik et al. (2013) have reported that β-glucans derived from *S. cerevisiae* alleviated clinical symptoms of inflammatory bowel syndrome in dogs by lowering the canine inflammatory bowel disease activity index, decreasing levels of the pro-inflammatory cytokine IL-6, and increasing anti-inflammatory IL-10 concentrations. However, few studies have focused on the effect of β-glucans on the canine microbial community.

Therefore, the aim of this work was to assess the potential beneficial effect of a *S. cerevisiae*-based product, characterized by high β-glucan and MOS content, in the modulation of the canine gut microbiota and fermentative metabolites, using an in vitro simulation of the gastrointestinal tract of dogs.

## Materials and methods

### Chemicals and test product

All chemicals were obtained from Sigma-Aldrich (Overijse, Belgium) unless stated otherwise. The test product BIOLEX MB 40 (Leiber GmbH, Bramsche, Germany) consisted of 100% of the cell walls of *S. cerevisiae* and was characterized by 27.5% β-glucans and 22.5% MOS. The remaining content of the test product included 25% raw protein, 7.5% crude fat, and other remaining cell wall components with a 6% moisture content. The *S. cerevisiae*-based product was tested at three different dosages, i.e., 0.5, 1.0, and 2.0 g/d, which would correspond to the in vivo recommendation of 0.05 to 0.2 % of the product in pet food and livestock feeds.

### Simulator of the canine intestinal microbial ecosystem

The reactor configuration was adapted from the Simulator of the Canine Intestinal Microbial Ecosystem (**SCIME**; Duysburgh et al., 2020), in order to allow the study of three different test concentrations of the *S. cerevisiae*-based test product in one single setup. In the current study, the reactor setup of the SCIME consisted of three different segments in order to test the three different test concentrations on parallel, with each segment consisting of a succession of three reactors simulating the different regions of the gastrointestinal tract, i.e., upper gastrointestinal tract including subsequent simulation of stomach and small intestine, proximal colon (**PC**), and distal colon (**DC**), respectively. Inoculum preparation, retention times, pH, temperature settings, and reactor feed composition were previously described (Duysburgh et al., 2020). The simulated nutritional medium, composed of 9 g/L dog food (Hill’s Science plan adult advance Fitness Lamb and Rice), 4 g/L mucin, 0.5 g/L cystein, 4 g/L special peptone (Oxoid, Aalst, Belgium), and 1.5 g/L yeast extract (Oxoid, Aalst, Belgium), was supplied to the model twice per day, representing a two meals per day feeding regime. Upon inoculation of each PC and DC with the microbiota derived from fresh fecal matter obtained from a Beagle dog, a 2-wk stabilization period was initiated to allow the microbial community to differentiate in the different reactors depending on the local environmental conditions, followed by a 2-wk control period to determine the baseline microbial community composition and activity. Following the control period, the *S. cerevisiae*-based test product was administered during each feeding cycle on top of the simulated nutritional medium in order to reach a daily dose of 0.5, 1.0, or 2.0 g/d over a 3-wk treatment period. Additionally, the SCIME setup was modified by incorporating a mucosal environment (the so-called M-SCIME), which takes into account the colonization of the mucus layer, as was previously reported for the human model (Van den Abbeele et al., 2012).

### Short-term colonic incubations

Given the regular flushing with N_2_ in the SCIME model to ensure anaerobiosis, gas production cannot be monitored in the long-term setup. Therefore, short-term colonic incubations were performed to evaluate total gas production. The colonic incubations were performed in closed penicillin bottles during which a single dose of the *S. cerevisiae*-based test product was supplied to a PC microbiota derived from the long-term SCIME during the control period. At the start of the colonic incubation, a sugar-depleted background medium (5.2 g/L K_2_HPO_4_; 16.3 g/L KH_2_PO_4_; 2.0 g/L NaHCO_3_; 2.0 g/L yeast extract; 2.0 g/L peptone; 1.0 g/L mucin; 0.5 g/L l-cysteine HCl; 2.0 mL/L Tween 80) was added to the penicillin bottles, which was made anaerobic by flushing with N_2_. To this background medium, a dose corresponding to 0.5, 1.0, and 2.0 g/d of the *S. cerevisiae*-based test product was supplemented separately. The negative control consisted of a similar incubation without the addition of the *S. cerevisiae*-based test product. Incubations were performed during 48 h, at 39 °C, and under continuous shaking (90 rpm). All experiments were performed in triplicate. Total gas production (handheld pressure indicator CPH6200; Wika, Echt, the Netherlands) was measured after 0, 6, 24, and 48 h of colonic incubation. pH measurements (Senseline F410; ProSense, Oosterhout, the Netherlands) were performed at the start of the colonic incubation and after 24 and 48 h.

### Microbial metabolic activity

During the control and treatment period of the SCIME experiment, samples for microbial metabolic activity were collected three times per week from each PC and DC. As described by De Weirdt et al. (2010), short-chain fatty acid (**SCFA**) production, including acetate, propionate, butyrate, and branched-chain fatty acids (**BCFA**) (isobutyrate, isovalerate, and isocaproate), was monitored. A commercially available enzymatic assay kit (R-Biopharm, Darmstadt, Germany) was used to determine lactate concentrations according to the manufacturer’s instructions. Ammonium analysis was conducted as described previously (Van de Wiele et al., 2004).

### Microbial community analysis

At the end of the control and treatment period of the SCIME experiment, samples for microbial community analysis were collected from the luminal and mucosal compartment of each PC and DC vessel. DNA was isolated as formerly reported (Vilchez-Vargas et al., 2013), starting from 1 mL luminal sample or 0.1 g mucosal sample. The microbiota profiling of each colon compartment was established both at the luminal and at the mucosal level. The 16S rRNA gene V3-V4 hypervariable regions were amplified by PCR using primers 341F (5′-CCT ACG GGN GGC WGC AG-3′) and 785Rmod (5′-GAC TAC HVG GGT ATC TAA KCC-3′), with the reverse primer being adapted from Klindworth et al. (2013) to increase coverage. The original genomic DNA extracts were diluted in DNase/RNase/protease-free water to obtain a concentration of 50 ng/µL, and 30 µL was sent out to LGC genomics GmbH (Germany) for library preparation and sequencing on an Illumina Miseq platform with v3 chemistry with the primers mentioned above.

### Statistics

Statistical analysis was performed in GraphPad Prism 8.3.0. Normality of data and equality of the variances were determined with a Shapiro–Wilk test and a Brown–Forsythe test, respectively. For normally distributed data with equal variances, an ANOVA with a Tukey post hoc test was used. For normally distributed data with unequal variances, a Welch test with a Games–Howell post hoc test was conducted. For non-normally distributed data, a Kruskal–Wallis ANOVA test with multiple post hoc pairwise comparisons was performed. Multiplicity adjusted *P*-values were implemented. In terms of statistics, the differences for all data discussed and indicated by *P* < 0.05 were significant with a confidence interval of 95%.

For the 16S-targeted Illumina data, read assembly and cleanup were largely derived from the MiSeq procedure as previously described (Schloss and Westcott, 2011; Kozich et al., 2013; De Paepe et al., 2017). In brief, assembly of reads into contigs, performance of alignment-based quality filtering, elimination of chimeras, assignment of taxonomy using a naïve Bayesian classifier (Wang et al., 2007) and RDP release 14 (Cole et al., 2009), and clustering of contigs into operational taxonomic units (**OTU**s) at 97% sequence similarity were performed using mothur (v. 1.39.5). All sequences classified as Eukaryota, Archaea, Chloroplasts, and Mitochondria were removed, as were sequences that could not be classified. For each OTU representative, sequences were picked as the most abundant sequence within that OTU.

## Results

### Analysis of overall fermentative activity

Gas production is a measure of microbial activity, with changes in gas production being indicative for the overall fermentation of the *S. cerevisiae*-based test product. Treatment with the *S. cerevisiae*-based test product increased gas production during all time intervals, with the most pronounced effect observed during the first 6 h of incubation (Table 1). Overall, the highest gas production was observed for the 2.0 g/d dose (i.e., 40.2 kPa after 48 h), reaching significantly higher values as compared with the other test conditions (*P* < 0.0001, *P* = 0.0003, and *P* = 0.0024 as compared with the blank, 0.5, and 1.0 g/d test condition, respectively). The increased gas production upon supplementation of the *S. cerevisiae*-based test product at a concentration of 2.0 g/d was accompanied by a pH decrease of 0.09 pH-units during the first 24 h of incubation, though not reaching statistical significance as compared with the control incubation (Table 1). Also for the other doses tested, acidification was not significantly different from the control incubation.

**Table 1.** Overall metabolic activity in terms of acidification and gas production^1^

Parameter	Time, h	Blank	0.5 g/d	1 g/d	2 g/d
Gas pressure, pKa	∆ 0 to 6	13.0^a^	14.9^b^	16.1^c^	17.9^d^
		(0.5)	(0.2)	(0.3)	(0.3)
	∆ 6 to 24	13.3^a^	14.9^a,b^	14.9^a^	16.5^b^
		(0.6)	(0.9)	(0.4)	(0.5)
	∆ 24 to 48	4.0^a^	4.5^a,b^	5.0^a,b^	5.8^b^
		(0.3)	(1.2)	(0.3)	(0.5)
pH	∆ 0 to 24	−0.05^a^	−0.06^a^	−0.04^a^	−0.09^a^
		(0.01)	(0.02)	(0.03)	(0.01)
	∆ 24 to 48	−0.05^a^	−0.04^a^	−0.04^a^	−0.04^a^
		(0.01)	(0.01)	(0.01)	(0.01)

^1^pH change between 0 and 24 and 24 and 48 h and increase in gas pressure (kPa) between 0 and 6, 6 and 24, and 24 and 48 h (±stdev) upon fermentation of three different concentrations of the *S. cerevisiae*-based test product (0.5, 1, and 2 g/d) by the canine fecal microbiota vs. the respective blank negative control (*n* = 3). The different test conditions were statistically compared for each experimental parameter and time point and statistical differences were indicated with different letters (*P* < 0.05).

### Analysis of the microbial metabolic activity

**Figure 1.**
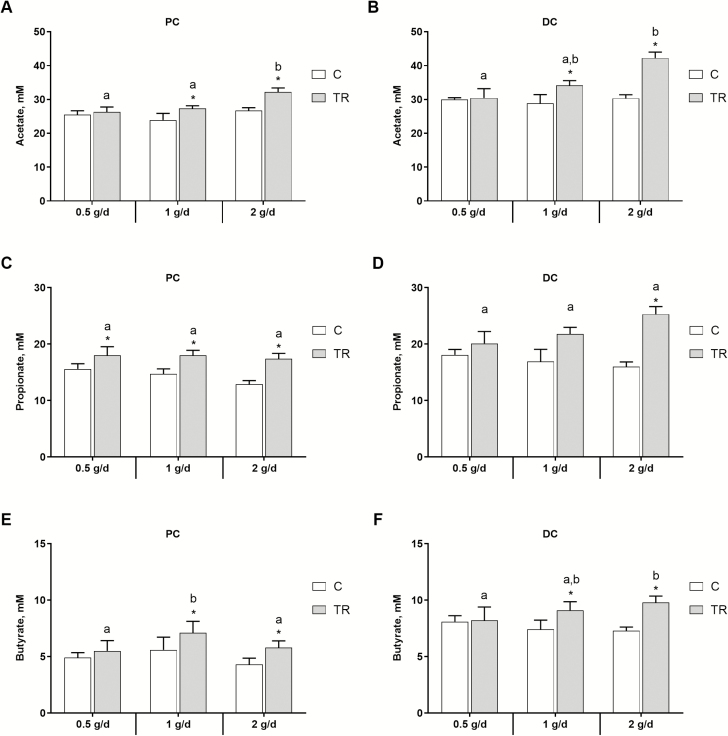
Microbial metabolic activity in terms of SCFA production. Average acetate (A&B; mM), propionate (C&D; mM), and butyrate (E&F; mM) production over the control (C; *n* = 6) and the treatment (TR; *n* = 9) period in the PC (left) and DC (right) of the canine gastrointestinal tract. Data are presented as mean ± stdev. Statistically significant differences relative to the control period are indicated with *, whereas significant differences among the test conditions are indicated with a different letter (*P* < 0.05).

A dose-dependent fermentation was observed when looking at the SCFA profiles, which consisted mainly of acetate, propionate, and butyrate (Figure 1) and small amounts of BCFA (Table 2). More specifically, supplementation of 1.0 and 2.0 g/d of the *S. cerevisiae*-based test product resulted in statistically significant increases in acetate production compared with the control period in both PC (*P* = 0.0003 and *P* < 0.0001, respectively) and DC (*P* = 0.0372 and *P* = 0.0019, respectively), whereas no significant effect could be observed for the 0.5 g/d dose. Moreover, the strongest effect was observed for the highest administration dose with an increased production of acetate of 5.5 mM (+ 21%) in PC and 11.9 mM (+ 39%) in DC, reaching significance in the PC (*P* < 0.0001). Similar observations were made for butyrate production, with significant increases being observed for the 1.0 and 2.0 g/d treatments as compared with the control period in both PC (*P* = 0.0253 and *P* = 0.0150, respectively) and DC (*P* = 0.0025 and *P* < 0.0001, respectively). In the DC, the butyrogenic effect was most pronounced upon supplementation of 2.0 g/d of the *S. cerevisiae*-based test product (+2.5 mM [+34%]), thereby reaching significantly higher values as compared with the lowest administration dose (*P* = 0.0013). As for propionate production, a statistically significant effect was observed for the three administered dosages of the *S. cerevisiae*-based test product in the PC (*P* = 0.0019 for the 0.5 g/d and *P* < 0.001 for the 1.0 and 2.0 g/d test dose), while in the DC the only significant propiogenic effect was observed upon dosing 2.0 g/d of the *S. cerevisiae*-based test product (*P* < 0.0001), i.e., an increase with 9.3 mM (+58%). Overall, BCFA levels were low (Table 2), with a significantly increased production observed upon dosing 1.0 g/d of the *S. cerevisiae*-based test product in both PC (*P* = 0.0386) and DC (*P* = 0.0019) and upon dosing 2.0 g/d in the DC (*P* = 0.0033).

**Table 2.** Microbial metabolic activity in terms of proteolytic markers^1^

		0.5 g/d		1 g/d		2 g/d	
Parameter	Colon region	C	TR	C	TR	C	TR
BCFA, mM	PC	1.48	2.34^a^	2.08	**3.27** ^a^	2.00	2.79^a^
		(0.44)	(1.10)	(1.13)	(0.42)	(0.51)	(0.30)
	DC	3.9	3.94^a^	3.59	**4.16** ^a,b^	3.78	**4.32** ^b^
		(0.14)	(0.41)	(0.37)	(0.15)	(0.16)	(0.17)
NH_4_^+^, mg/L	PC	252	258^a^	265	281^a,b^	211	**317** ^b^
		(19)	(38)	(22)	(65)	(42)	(22)
	DC	446	422^a^	424	439^a,b^	375	**469** ^b^
		(34)	(36)	(18)	(45)	(9)	(30)

^1^Average ammonium (NH4+; mg/L) and BCFA (mM) production over the control (C; *n* = 6) and the treatment (TR; *n* = 9) period in the PC and DC of the canine gastrointestinal tract. Data are presented as mean (±stdev). Statistically significant differences relative to the control period are indicated in bold, whereas significant differences between the test conditions are indicated with a different letter (*P* < 0.05).

Ammonium production remained unaffected, except for a significant increase in the PC (*P* = 0.0002) and DC (*P* < 0.0001) upon treatment with the 2.0 g/d dose (Table 2).

**Figure 2.**
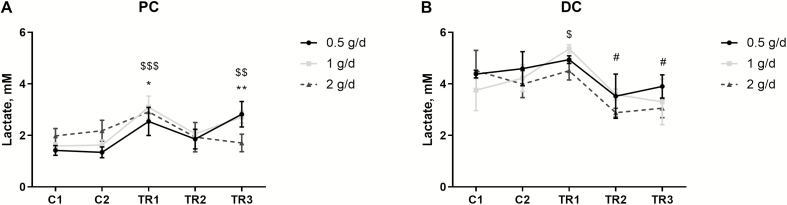
Microbial metabolic activity in terms of lactate production. Average lactate (mM) production over two control weeks (C1 and C2; *n* = 3 per week) and three treatment weeks (TR1, TR2, and TR3; *n* = 3 per week) in the PC (left) and DC (right) of the canine gastrointestinal tract. Data are presented as mean ± stdev. Statistically significant differences relative to C1 are indicated with *, $, # for 0.5, 1, and 2 g/d, respectively (*/$/# for 0.05 < *P* < 0.01; **/$$/## for 0.01 < *P* < 0.001; ***/$$$/### for 0.001 < *P* < 0.0001).

Finally, differences in lactate levels were observed during the treatment with different dosages of the *S. cerevisiae*-based test product (Figure 2). Upon dosing 0.5 and 1.0 g/d, a significant increase in lactate concentrations was observed in the PC at the end of the treatment period as compared with the control period (*P* = 0.0095 and *P* = 0.0020, respectively), whereas in the DC, the test dose of 2.0 g/d resulted in significantly reduced lactate levels at the end of the treatment period (*P* = 0.0294).

### Analysis of the microbial community composition

As the three SCIME units were identical prior to treatment, starting communities were characterized to compare differences between luminal and mucosal microbiota in both PC and DC. A phylum-specific colonization of the luminal vs. the mucosal compartment was observed (Table 3). Actinobacteria were significantly enriched in the mucosal environment in both PC and DC, with the main colonization of these phyla occurring in the PC. On the other hand, Firmicutes abundance was significantly enhanced in the luminal PC and DC. Further, tendencies (*P* > 0.1) to lower abundances in the mucus layer for Bacteroidetes and Proteobacteria in PC and Fusobacteria in DC were observed. As for the latter phylum, strongly increased abundances were observed in the DC as compared with the PC in the luminal environment (*P* = 0.0101). Also a family-specific colonization of the lumen vs. the mucus layer was observed in the microbial community of the SCIME prior to treatment (Table 3). In both PC and DC, Bifidobacteriaceae specifically colonized the mucosal environment, whereas Veillonellaceae preferred the luminal environment, with the main colonization of these families occurring in the PC. Additionally, for families mainly colonizing the DC, abundances of Acidaminococcaceae and Pseudomonadaceae were higher in the luminal compartment, whereas Erysipelotrichaceae, Lachnospiraceae, and Sutterellaceae were enriched in the mucus layer.

**Table 3.** Region-specific colonization of starting community^1^

		Abundance, %				*P*-value			
		L		M		L vs. M		PC vs. DC	
Phylum	Family	PC	DC	PC	DC	PC	DC	Lumen	Mucus
Actinobacteria	Bifidobacteriaceae	2.5	1.1	53.9	25.6	0.0156	0.0360		0.0375
	Coriobacteriaceae	0.0	0.0	0.8	1.4				
	Total	2.5	1.1	54.7	26.9	0.0143	0.0245		0.0372
Bacteroidetes	Bacteroidaceae	15.7	13.7	3.3	13.2				
	Porphyromonadaceae	0.5	6.4	1.5	10.6				
	Prevotellaceae	2.6	4.0	1.0	0.9				
	Total	18.7	24.1	5.8	24.8				0.0283
Firmicutes	Acidaminococcaceae	0.4	0.9	0.2	0.4		0.0577		
	Clostridiaceae_1	0.0	0.0	0.1	4.0				
	Clostridiales_unclassified	0.0	0.1	0.0	1.0				
	Enterococcaceae	0.0	0.0	0.0	0.0				
	Erysipelotrichaceae	0.4	0.1	3.3	2.1		0.0246		
	Lachnospiraceae	2.4	2.5	3.6	10.7		0.0176		0.0842
	Lactobacillaceae	1.7	0.1	3.1	0.2				
	Peptococcaceae_1	0.0	0.0	0.2	1.7				
	Ruminococcaceae	0.2	0.2	4.7	4.6				
	Veillonellaceae	61.0	49.9	22.6	11.5	0.0034	0.0224	0.0128	0.0791
	Total	66.2	53.9	38.0	36.4	0.0189	0.0243		
Fusobacteria	Fusobacteriaceae	0.0	14.9	0.0	7.0			0.0101	
Proteobacteria	Enterobacteriaceae	11.0	2.8	0.2	0.4			0.0913	
	Pseudomonadaceae	1.2	2.2	0.0	0.1		0.0936	0.0205	
	Succinivibrionaceae	0.0	0.0	0.4	1.7				
	Sutterellaceae	0.0	1.0	0.7	2.7		0.0833	0.0994	
	Xanthomonadaceae	0.2	0.0	0.0	0.0				
	Total	12.5	6.0	1.5	5.0				

^1^Average abundance (%) at microbial phylum and family level in the lumen (L) and mucus (M) of the PC and DC of three units of the SCIME at the end of the control period (*n* = 3). Further, also significant differences for a certain family between L and M or between PC and DC are indicated by means of their *P*-value (*P* < 0.05), as calculated using an ANOVA with post hoc Tukey test for multiple comparisons. Differences that were statistically different are indicated by dark gray shading (*P* < 0.05), while differences that tended to be statistically different are indicated by light gray shading (0.05 < *P* < 0.10).

**Figure 3.**
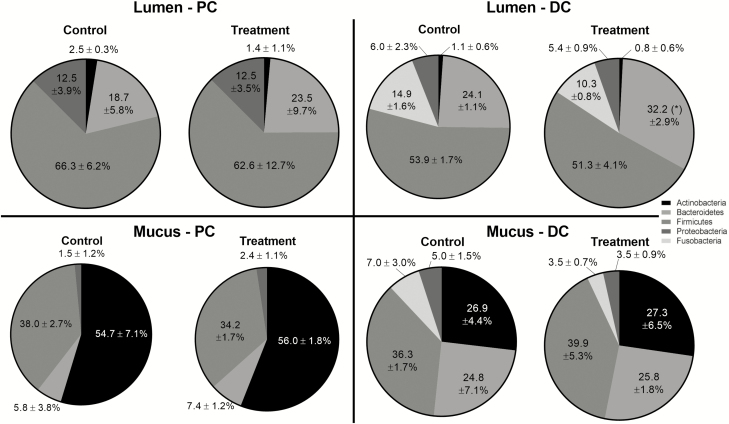
Microbial community composition as assessed via 16S-targeted Illumina sequencing. Abundance (%) at microbial phylum level in the lumen and mucus of the PC and DC of the canine gastrointestinal tract over the control (C) and the treatment (TR) period. For optimal observation of consistent effects for the different concentrations tested (0.5, 1, and 2 g/d), the average (±stdev) of the three test concentrations is presented (*n* = 3). Statistically significant differences relative to the control period are indicated with * (*P* < 0.05).

Upon treatment with the *S. cerevisiae*-based test product, a consistent increase in the Bacteroidetes phylum was observed in the luminal compartment in both PC and DC (Figure 3), reaching significance in the DC (*P* = 0.0067). In the DC, this increase was mainly at the expense of the Fusobacteria phylum. At mucosal level, no significant changes were observed upon treatment with the *S. cerevisiae*-based test product. At the family level, the strongest treatment effects were observed in the luminal (Table 4) and not the mucosal environment (Supplementary Table S1). The consistent increase of Bacteroidetes (phylum level) in the luminal compartment of the SCIME was related to changes in three families within this phylum. Indeed, consistent decreases in the abundance of Bacteroidaceae were observed in both PC and DC upon the addition of the *S. cerevisiae*-based test product. In contrast, Porphyromonadaceae and Prevotellaceae specifically increased upon supplementation of the *S. cerevisiae*-based test product. When further analyzing data at the lowest phylogenetic level (OTU level) within the Porphyromonadaceae family, it followed that two OTUs were mainly responsible for the observed increases. These two OTUs were related to *Parabacteroides merdae* (OTU5) and an unclassified Porphyromonadaceae species (OTU19). The main family belonging to the Firmicutes phylum was the Veillonellaceae family. This family remained unaffected upon supplementation of the *S. cerevisiae*-based test product. Other families within the Firmicutes phylum, such as Lactobacillaceae and Lachnospiraceae, decreased upon test product addition. On the other hand, the Enterococcaceae family consistently increased upon treatment with the *S. cerevisiae*-based test product in the PC, with the strongest increases observed for treatments with the 0.5 and 1.0 g/d doses. The main family belonging to the Proteobacteria was the Enterobacteriaceae family, which decreased in both PC and DC upon product supplementation. This effect was masked at the phylum level due to increases of other Proteobacteria families upon treatment, i.e., Pseudomonadaceae, Succinivibrionaceae, Sutterellaceae, and Xanthomonadaceae. The Actinobacteria phylum consisted of Bifidobacteriaceae and Coriobacteriaceae. A decrease in abundance of the Bifidobacteriaceae family was observed upon treatment with the *S. cerevisiae*-based test product. Finally, as was observed at the phylum level (Fusobacteria), treatment with the *S. cerevisiae*-based test product reduced the abundance of the Fusobacteriaceae family in the DC.

**Table 4.** Luminal microbial community composition at the family level^1^

		PC						DC					
		0.5 g/d		1.0 g/d		2 g/d		0.5 g/d		1.0 g/d		2 g/d	
Phylum	Family	C	TR	C	TR	C	TR	C	TR	C	TR	C	TR
Actinobacteria	Bifidobacteriaceae	2.8	2.2	2.2	1.8	2.7	0.2	0.7	1.4	0.9	0.8	1.8	0.2
Bacteroidetes	Bacteroidaceae	10.2	3.6	22.2	2.2	14.7	1.6	19.5	13.1	9.7	7.0	11.7	1.3
	Porphyromonadaceae	0.3	2.9	0.7	4.1	0.5	1.9	3.2	15.8	5.9	20.7	10.3	14.8
	Prevotellaceae	2.0	6.3	1.0	25.7	4.7	22.2	1.9	2.2	7.2	7.9	2.8	14.0
Firmicutes	Acidaminococcaceae	0.2	0.6	0.7	0.8	0.3	0.3	0.9	0.6	1.0	1.2	0.9	1.2
	Clostridiales_unclassified	0.0	0.0	0.0	0.0	0.0	0.0	0.0	0.0	0.1	0.1	0.1	0.0
	Enterococcaceae	0.0	0.2	0.0	0.1	0.0	0.0	0.0	0.0	0.0	0.0	0.0	0.0
	Erysipelotrichaceae	0.6	1.5	0.2	1.6	0.4	0.2	0.1	0.4	0.1	0.7	0.2	0.2
	Lachnospiraceae	5.0	1.6	0.9	0.9	1.5	0.6	2.1	1.5	2.6	2.2	2.9	1.1
	Lactobacillaceae	3.0	0.1	0.7	0.0	1.3	0.0	0.1	0.0	0.0	0.0	0.0	0.0
	Ruminococcaceae	0.2	0.1	0.3	0.1	0.2	0.1	0.2	0.1	0.3	0.2	0.3	0.2
	Veillonellaceae	63.9	72.3	62.2	47.8	57.1	58.6	51.7	49.5	50.5	42.4	47.5	52.1
Fusobacteria	Fusobacteriaceae	0.0	0.0	0.0	0.0	0.0	0.0	15.7	10.8	15.9	10.6	13.0	9.4
Proteobacteria	Enterobacteriaceae	11.2	1.6	6.9	2.3	15.1	3.2	1.6	0.3	1.9	0.6	4.9	1.3
	Pseudomonadaceae	0.5	5.5	1.8	11.5	1.3	9.6	1.5	2.6	2.9	4.4	2.1	2.4
	Succinivibrionaceae	0.0	0.7	0.1	0.3	0.0	0.0	0.0	0.0	0.0	0.0	0.0	0.0
	Sutterellaceae	0.0	0.2	0.1	0.5	0.0	0.1	0.7	1.3	1.0	1.3	1.4	1.4
	Xanthomonadaceae	0.1	0.3	0.1	0.1	0.3	1.2	0.0	0.3	0.0	0.0	0.0	0.3

^1^Abundances of the dominant families belonging to the Firmicutes, Proteobacteria, Actinobacteria, Bacteroidetes, and Fusobacteria phylum (%) as assessed via 16S-targeted Illumina sequencing, during the control (C) and treatment (TR) period in luminal samples of both the PC and DC for three different test concentrations (0.5, 1, and 2 g/d) (*n* = 1). The intensity of the shading indicates the absolute abundance, normalized for each of the different families (i.e., per row).

## Discussion

In this study, the effect of repeated dosage of three concentrations of a *S. cerevisiae*-based test product, characterized by 27.5% β-glucans and 22.5% MOS, was assessed in terms of modulation of the canine gut microbiota and fermentative metabolites using a standardized in vitro gut model using the fecal microbiota of a single dog.

By using the Illumina Miseq platform, the starting microbial community composition of the dog was analyzed in the in vitro SCIME model. At the phylum level, an average community composition of 60.1% Firmicutes, 21.4% Bacteroidetes, 9.3% Proteobacteria, 7.4% Fusobacteria, and 1.8% Actinobacteria was detected when including both the PC and DC compartments of the simulated canine gastrointestinal tract, which corresponded to the microbial profile recently reported by Duysburgh et al. (2020) using the same in vitro platform. Furthermore, a highly similar microbial community composition was obtained as was recently reported in vivo (Kim et al., 2017). Indeed, it was shown by using the Illumina Miseq platform that the canine microbial community composition is significantly affected by the type of diet, but that on average, it is composed of Firmicutes (64.2% to 73.3%), Bacteroidetes (17.3% to 19.9%), Proteobacteria (0.9% to 8.7%), Fusobacteria (0% to 13.6%), and Actinobacteria (0.6% to 1.5%) (Kim et al., 2017). The observed similarities with in vivo data provide a good starting point to assess the fermentation potential of the *S. cerevisiae*-based test product in the canine gastrointestinal tract using the in vitro SCIME model. Furthermore, microbial community development in the SCIME model proved to be colon-region specific, as was previously reported (Duysburgh et al., 2020). The PC was characterized by an increased abundance of Bifidobacteriaceae and Veillonellaceae, while the DC was specifically colonized by members of the Fusobacteria phylum. On the other hand, the mucosal environment proved to be essential for the growth of Bifidobacteriaceae, Erysipelotrichaceae, and Lachnospiraceae, the latter being similar as observed by Van den Abbeele et al. (2013) in the human Simulator of the Human Intestinal Microbial Ecosystem (SHIME) model. Altogether, these results suggest that the use of the in vitro SCIME model provides an accurate model to study the fermentation potential of different substrates in the canine gastrointestinal tract.

Upon product administration, a stimulation of acetate, propionate, and butyrate production was observed upon repeated supplementation of the *S. cerevisiae*-based test product in both the PC and DC, with the strongest effects observed for the highest dose tested. A consistent finding for all three tested concentrations was the significantly increased propionate production in the PC. It has been shown previously that the addition of MOS, derived from the yeast cell wall of *S. cerevisiae*, to the diet tended to increase ileal propionate levels in adult dogs (Strickling et al., 2000). In dogs, propionate production has been shown to play a role in satiety through the stimulation of gastrointestinal satiety hormones, such as Glucagon-like peptide-1 (GLP-1) and Peptide YY. Massimino et al. reported that secretion of GLP-1 by enteroendocrine L-cells, which are predominantly present in the distal part of the canine gastrointestinal tract (Holst, 2007), was enhanced upon supplementation of fermentable dietary fibers in healthy dogs (Massimino et al., 1998). In the current study, the stimulation of propionate correlated with the increased abundance of the Bacteroidetes phylum. Application of 16S-based Illumina sequencing allowed to point out that increases within this phylum were related to increases in the Porphyromonadaceae and Prevotellaceae, two families containing potent propionate producers (Sakamoto, 2014; Krieg, 2011).

With respect to other saccharolytic metabolites, treatment with lower concentrations of the *S. cerevisiae*-based test product resulted in initially higher lactate concentrations, indicating the stimulation of lactate-producing substrate degraders. Indeed, it has been shown that fermentation of MOS by the canine microbiota increases lactate concentrations in vitro, suggesting the ability of lactate-producing species to utilize MOS as a primary substrate (Hussein and Healy, 2001). Application of molecular tools showed that likely Enterococcaceae were stimulated as main lactate producers (as opposed to *Lactobacilli* and *Bifidobacteria*). Kim et al. (2017) have shown that Enterococcaceae are indeed part of the core microbiome of dogs, whereas Bifidobacteriaceae and Lactobacillaceae are not. Lactate is considered as a health-promoting metabolite in the gut, as it exerts strong antimicrobial effects against pathogens (Alakomi et al., 2000; Raybaudi-Massilia et al., 2009). Besides the antipathogenic activity, lactate can contribute to health by its conversion into butyrate through cross-feeding interactions (Bourriaud et al., 2005). In the current study, this was observed for the highest dose tested (2.0 g/d), where lactate levels significantly decreased during the final weeks of treatment in the DC, thereby probably contributing to the increased levels of butyrate.

Other consistent changes in community composition upon product supplementation included the decrease in the Enterobacteriaceae and the Fusobacteriaceae families, which both contain several potentially opportunistic pathogens. Several Enterobacteriaceae species are known to cause disease in dogs, including amongst others *E. coli*, which is a common cause of urinary tract infections in dogs (Sykes, 2013). Previous studies have shown that supplementation of the spray-dried yeast cell wall to the diet of adult dogs decreases fecal *E. coli* concentrations in a dose-dependent manner (Middelbos et al., 2007).

Additionally, increased SCFA production in the DC confirms that a proportion of the product had been able to withstand fermentation in the PC and was transferred to the DC. Calabrò et al. (2013) have shown that supplementation of the dried yeast cell wall to the diet promoted a more distal fermentation in the canine gastrointestinal tract in vitro. As saccharolytic fermentation mainly takes place in the proximal regions of the colon, fermentation in the DC is mainly proteolytic (Williams et al., 2001). Considering that proteolytic fermentation can lead to the formation of potentially toxic metabolites, such as indoles and ammonia, there is great interest in finding substrates with biological activity in the DC (Terpend et al., 2013). Although markers of proteolytic fermentation (ammonium and BCFA) significantly increased in the DC compartment in the current study, probably due to the addition of proteins that were contained in the *S. cerevisiae*-based test product (i.e., protein content of 25%), the distal production of SCFA was an interesting finding in this perspective.

A potential shortcoming of the study design includes the absence of analysis of interindividual effects among different dogs in response to treatment with the *S. cerevisiae*-based test product. Indeed, in the current study, fecal material of one Beagle dog was used as microbial inoculum for the in vitro platform as the aim was to investigate potential gut-modulatory effects of the *S. cerevisiae*-based test product and not to address donor-dependent responses to the treatment. However, it has been reported that the composition of the intestinal microbiota among dogs can vary considerably (Kim et al., 2017) and, therefore, might result in different responses to dietary intervention. The inclusion of different animal donors might have provided further insight into the effect of the *S. cerevisiae*-based test product on the canine microbial community.

From this study, it can be concluded that repeated dosage of a product high in β-glucans and MOS affected the gut microbial activity and composition in the canine gastrointestinal tract in vitro. Changes in terms of metabolic activity could be linked to specific microbial alterations at the family level, such as the specific stimulation of the propionate-producing families Porphyromonadaceae and Prevotellaceae, upon product supplementation. The generated data support a possible health-promoting role of the *S. cerevisiae*-based test product when supplemented to the dogs’ diet. Further research is warranted to confirm these findings.

## Supplementary Material

skaa173_suppl_Supplementary_Table_S1

## Abbreviations

BCFA: branched-chain fatty acid
DC: distal colon
MOS: mannan-oligosaccharides
OTU: operational taxonomic unit
PC: proximal colon
SCFA: short-chain fatty acid
SCIME: Simulator of the Canine Intestinal Microbial Ecosystem
