# Supplementary material for: Dried yeast cell walls high in beta-glucan and mannan-oligosaccharides positively affect microbial composition and activity in the canine gastrointestinal tract in vitro
Source: J Anim Sci. 2020 Jun 4;98(6):skaa173. doi: 10.1093/jas/skaa173 (PMC7295327; doi:10.1093/jas/skaa173)
Supplement: skaa173_suppl_Supplementary_Table_S1 [file skaa173_suppl_supplementary_table_s1.docx]

**SUPPLEMENTARY INFORMATION**

**DRIED YEAST CELL WALLS HIGH IN BETA-GLUCAN AND MANNAN-OLIGOSACCHARIDES POSITIVELY AFFECT MICROBIAL COMPOSITION AND ACTIVITY IN THE CANINE GASTROINTESTINAL TRACT IN VITRO**

Pieter Van den Abbeele^*^, Cindy Duysburgh^*^, Maike Rakebrandt^$^, and Massimo Marzorati^*,#^

^*^ ProDigest bvba, Technologiepark 82, 9052 Ghent, Belgium; ^$^ Leiber GmbH, Hafenstrasse 24, 49565 Bramsche, Germany ; ^#^ Center of Microbial Ecology and Technology (CMET), Ghent University, Coupure Links 653, 9000 Ghent, Belgium

Running title: *In vitro* effect of yeast cell walls in dogs

Corresponding author: Massimo Marzorati, Phone: +3292411190, Fax: +3292411191, E-mail: [massimo.marzorati@prodigest.eu](mailto:massimo.marzorati@prodigest.eu)

**Table 1SI. Mucosal microbial community composition at family level.** Abundances of the dominant families belonging to the Firmicutes, Proteobacteria, Actinobacteria, Bacteroidetes and Fusobacteria phylum (%) as assessed via 16S-targeted Illumina sequencing, during the control (C) and treatment (TR) period in mucosal samples of both the proximal (PC) and distal colon (DC) for three different test concentrations (0.5 g/day, 1 g/day and 2 g/day) (n = 1). The intensity of the shading indicates the absolute abundance, normalized for each of the different families (i.e. per row).

| **Phylum** | **Family** | **PC** | | | | | | **DC** | | | | | |
| --- | --- | --- | --- | --- | --- | --- | --- | --- | --- | --- | --- | --- | --- |
|  |  | **0.5 g/day** | | **1.0 g/day** | | **2 g/day** | | **0.5 g/day** | | **1.0 g/day** | | **2 g/day** | |
|  |  | **C** | **TR** | **C** | **TR** | **C** | **TR** | **C** | **TR** | **C** | **TR** | **C** | **TR** |
| **Actinobacteria** | ***Bifidobacteriaceae*** | 53.3 | 56.6 | 46.9 | 55.7 | 61.4 | 52.1 | 30.2 | 26.4 | 20.1 | 32.5 | 26.4 | 19.5 |
|  | ***Coriobacteriaceae*** | 0.3 | 0.9 | 1.3 | 0.7 | 0.8 | 1.9 | 0.6 | 1.2 | 2.0 | 1.0 | 1.5 | 1.1 |
| **Bacteroidetes** | ***Bacteroidaceae*** | 4.0 | 2.0 | 4.8 | 4.1 | 1.2 | 1.9 | 18.3 | 9.7 | 10.2 | 12.7 | 11.2 | 10.1 |
|  | ***Porphyromonadaceae*** | 0.6 | 2.8 | 3.4 | 0.7 | 0.5 | 4.1 | 4.6 | 14.8 | 22.0 | 11.4 | 5.2 | 16.4 |
|  | ***Prevotellaceae*** | 1.5 | 2.2 | 1.4 | 1.7 | 0.1 | 2.7 | 0.3 | 0.4 | 0.3 | 0.6 | 2.1 | 1.2 |
| **Firmicutes** | ***Acidaminococcaceae*** | 0.2 | 0.1 | 0.2 | 0.2 | 0.1 | 0.6 | 0.2 | 0.2 | 0.4 | 0.2 | 0.5 | 0.6 |
|  | ***Clostridiaceae_1*** | 0.2 | 0.0 | 0.0 | 0.0 | 0.1 | 0.0 | 7.3 | 0.0 | 0.1 | 0.8 | 4.6 | 0.3 |
|  | ***Clostridiales_unclassified*** | 0.0 | 0.1 | 0.1 | 0.0 | 0.0 | 1.7 | 1.0 | 0.3 | 0.4 | 2.7 | 1.7 | 0.5 |
|  | ***Enterococcaceae*** | 0.0 | 0.0 | 0.1 | 0.0 | 0.0 | 0.0 | 0.0 | 0.0 | 0.1 | 0.0 | 0.0 | 0.1 |
|  | ***Erysipelotrichaceae*** | 0.9 | 16.5 | 3.9 | 0.8 | 5.1 | 2.9 | 2.3 | 18.5 | 2.3 | 0.7 | 1.8 | 9.3 |
|  | ***Lachnospiraceae*** | 3.1 | 1.6 | 5.6 | 4.1 | 2.1 | 7.1 | 8.9 | 10.9 | 11.3 | 10.5 | 11.9 | 12.0 |
|  | ***Lactobacillaceae*** | 5.8 | 0.0 | 0.8 | 6.1 | 2.7 | 0.7 | 0.3 | 0.0 | 0.0 | 0.1 | 0.2 | 0.1 |
|  | ***Peptococcaceae_1*** | 0.0 | 0.9 | 0.1 | 0.0 | 0.5 | 0.0 | 1.7 | 0.2 | 0.5 | 10.3 | 3.0 | 0.3 |
|  | ***Ruminococcaceae*** | 6.2 | 1.8 | 1.7 | 3.5 | 6.1 | 1.4 | 3.5 | 2.0 | 2.8 | 3.8 | 7.4 | 4.0 |
|  | ***Veillonellaceae*** | 23.2 | 11.3 | 26.6 | 21.0 | 17.9 | 19.4 | 9.1 | 9.0 | 19.4 | 4.5 | 6.0 | 17.0 |
| **Fusobacteria** | ***Fusobacteriaceae*** | 0.0 | 0.0 | 0.0 | 0.0 | 0.0 | 0.0 | 7.5 | 3.6 | 3.7 | 4.1 | 9.6 | 2.8 |
| **Proteobacteria** | ***Enterobacteriaceae*** | 0.4 | 0.3 | 0.1 | 0.6 | 0.3 | 0.3 | 0.3 | 0.1 | 0.4 | 0.1 | 0.5 | 0.5 |
|  | ***Pseudomonadaceae*** | 0.0 | 0.2 | 0.0 | 0.0 | 0.0 | 0.0 | 0.1 | 0.0 | 0.1 | 0.1 | 0.2 | 0.1 |
|  | ***Succinivibrionaceae*** | 0.0 | 0.2 | 0.9 | 0.0 | 0.4 | 0.3 | 1.8 | 0.6 | 0.5 | 0.4 | 2.8 | 1.7 |
|  | ***Sutterellaceae*** | 0.1 | 2.2 | 1.8 | 0.4 | 0.4 | 2.4 | 1.8 | 1.8 | 3.2 | 3.0 | 3.2 | 2.0 |
|  | ***Xanthomonadaceae*** | 0.0 | 0.0 | 0.1 | 0.0 | 0.0 | 0.1 | 0.0 | 0.0 | 0.1 | 0.0 | 0.0 | 0.1 |
